# Induction of Autophagy to Achieve a Human Immunodeficiency Virus Type 1 Cure

**DOI:** 10.3390/cells10071798

**Published:** 2021-07-16

**Authors:** Grant R. Campbell, Stephen A. Spector

**Affiliations:** 1 Division of Infectious Diseases, Department of Pediatrics, University of California San Diego, La Jolla, CA 92093, USA; saspector@ucsd.edu; 2 Rady Children’s Hospital, San Diego, CA 92123, USA

**Keywords:** HIV-1, autophagy, latency promoting approach, cell death, nanoparticle, SMAC mimetics, apoptosis, autosis

## Abstract

Effective antiretroviral therapy has led to significant human immunodeficiency virus type 1 (HIV-1) suppression and improvement in immune function. However, the persistence of integrated proviral DNA in latently infected reservoir cells, which drive viral rebound post-interruption of antiretroviral therapy, remains the major roadblock to a cure. Therefore, the targeted elimination or permanent silencing of this latently infected reservoir is a major focus of HIV-1 research. The most studied approach in the development of a cure is the activation of HIV-1 expression to expose latently infected cells for immune clearance while inducing HIV-1 cytotoxicity—the “kick and kill” approach. However, the complex and highly heterogeneous nature of the latent reservoir, combined with the failure of clinical trials to reduce the reservoir size casts doubt on the feasibility of this approach. This concern that total elimination of HIV-1 from the body may not be possible has led to increased emphasis on a “functional cure” where the virus remains but is unable to reactivate which presents the challenge of permanently silencing transcription of HIV-1 for prolonged drug-free remission—a “block and lock” approach. In this review, we discuss the interaction of HIV-1 and autophagy, and the exploitation of autophagy to kill selectively HIV-1 latently infected cells as part of a cure strategy. The cure strategy proposed has the advantage of significantly decreasing the size of the HIV-1 reservoir that can contribute to a functional cure and when optimised has the potential to eradicate completely HIV-1.

## 1. Introduction

Within days of infection, human immunodeficiency virus type 1 (HIV-1) rapidly disseminates to draining lymph nodes. HIV-1 advances to destroy helper T cells and establish latent HIV-1 reservoirs through the irreversible integration of replication-competent proviral DNA into the genome of resting CD4^+^ T cells, hematopoietic stem cells, cells from the monocyte-macrophage lineage, and microglia [1,2,3,4,5]. Although antiretroviral therapy (ART) has transformed HIV-1 infection from a lethal and destructive disease into a controllable, but chronic condition, the establishment of HIV-1 latency is unaffected by early initiation of ART [6], and there is no evidence that ART alone is able to eradicate latent HIV-1 [7,8]. Moreover, although ART effectively suppresses HIV-1 replication, persistent residual low-level viremia maintains the latent pool [9,10], and the very low expression of viral proteins enables latently infected cells to evade detection and clearance by the immune system and undergo homeostatic proliferation [11,12,13,14,15]. Additionally, as viral loads rebound to high pre-therapy levels following ART interruption, albeit with significant heterogeneity in the speed of viral rebound (days, weeks, or sometimes years [16,17]), lifelong ART is required for continued viral suppression. Therefore, these latent cellular reservoirs present the major obstacle in preventing the eradication of HIV-1.

As with most viruses, HIV-1 must hijack and exploit host cell pathways for its replication and propagation, including the transcription and translation of viral proteins, and for the assembly of new virions. Biosynthesis within mammalian cells is a balance between anabolism and catabolism. While growth factors and cytokines regulate anabolism, conditions of stress promote catabolism under the regulation of AMPK (5′ AMP-activated protein kinase). Both anabolism- and catabolism-promoting signals converge at the evolutionarily conserved atypical serine/threonine kinase mechanistic target of rapamycin (MTOR) [18,19]. MTOR serves as a master regulator of cellular functions in response to diverse signals, including growth factors, immune signals, DNA damage, oxygen, metabolic cues, lysosomal activities, and infections. MTOR forms two structurally and functionally different complexes: MTOR complex 1 (MTORC1) and MTOR complex 2 (MTORC2). Although MTORC1 and MTORC2 are responsive to different signalling mechanisms and produce differing downstream effects, whereby the activation of either facilitates cell growth and survival [20,21]. MTORC2 functions include actin cytoskeleton organization, control of Na^+^ transport, and the inhibition of apoptosis [22,23,24], while MTORC1 targets downstream RPS6KB1, EIF4EBP1, and others to promote cellular growth and proliferation via increased protein and lipid synthesis, while repressing catabolic programmes resulting in the inhibition of autophagy. In relation to HIV-1-infection, the hyper-activation of MTORC1 by HIV-1 Tat is necessary for HIV-1 expression [25], and the integrity and function of both MTORC1 and MTORC2 are essential to re-activate HIV-1 from latency [26,27,28].

Autophagy is a catabolic intracellular lysosomal degradation pathway that ensures homeostasis through the elimination of misfolded protein aggregates, sub-cellular organelles, and microbial pathogens thereby promoting innate immunity and stimulating growth and/or survival [29,30,31,32,33,34]. Autophagy can be either non-selective—macroautophagy (hereafter referred to as autophagy) or selective. In selective autophagy, it mediates the degradation of specific cargo such as mitochondria (mitophagy), endoplasmic reticulum (reticulophagy), or invading microorganisms (xenophagy) [35,36]. In the latter, it also extensively interacts with immunometabolism to control infection and inflammation [37]. Both canonical and non-canonical autophagy pathways play pivotal roles in host defence and innate immune responses against HIV-1, and there are numerous reviews covering the mechanism, function, and regulation of both canonical and non-canonical autophagy during HIV-1 infection [38,39,40,41]. Here, we provide a brief summary of the potential role for autophagy as part of an HIV-1 cure strategy.

## 2. The HIV-1 Latent Reservoir

Although the mechanisms by which integrated HIV-1 proviral DNA becomes quiescent in vivo are not fully understood, hindering the development of effective curative strategies, the current understanding is that it is a multifactorial process that operates at the transcriptional and post-transcriptional levels to repress HIV-1 expression. Regardless of the integration site, the 5′ LTR of the transcriptionally silent provirus is embedded within nucleosomes (nuc) [42]. The short isoform of BRD4 recruits the cellular ATP-dependent chromatin remodelling BRG1/BRM-associated factor (BAF) complex to the proviral genome [43] where it actively positions nuc-1 in a refractory sequence immediately downstream of the HIV-1 transcription start site to generate a repressive chromatin conformation [44]. Although *cis*-regulatory elements located at the 5′ LTR strongly recruit cellular transcription factors and coactivators to initiate transcription, RNA polymerase II (RNAPII) undergoes promoter-proximal stalling at sites that overlap with nuc-1 after the production of a short nascent RNA transcript that includes the trans-activation response (TAR) element. The recruitment of the multi-subunit negative transcription elongation factors, NELF and DSIF, stabilise the paused RNAPII [45,46,47,48]. To overcome this, HIV-1 Tat binds the TAR element, where it undergoes K28 acetylation by the histone acetyltransferase (HAT) KAT2B, promoting the recruitment of positive transcription elongation factor b (P-TEFb; composed of CDK9 and CCNT1) and the super elongation complex [49,50,51,52,53]. The CDK9 subunit of P-TEFb phosphorylates NELF (which dissociates from TAR) [54], DSIF (converting it to an elongation factor) [55], and the RNAPII C-terminal domain, which unpauses and switches to a highly processive elongation complex [56]. HATs, including EP300 and KAT2A, then converge to acetylate HIV-1 Tat K50/51 [57]. The former facilitates the release of P-TEFb and the recruitment of the chromatin remodelling polybromo-associated BAF (PBAF) complex to the HIV-1 promoter to reposition nuc-1 downstream of the transcription start site [44,58]. The latter mediates the transfer of HIV-1 Tat from the TAR element to RNAPII promoting elongation [59]. Therefore, the availability and abundance of P-TEFb and HIV-1 Tat are crucial for HIV-1 transcription elongation. BCL11B represses P-TEFb recruitment through sequestration of P-TEFb in a complex containing 7SK small nuclear ribonucleoprotein (snRNP) and HEXIM1 thereby preventing efficient HIV-1 transcription elongation [60,61,62]. HIV-1 Vpr counteracts BCL11B-mediated viral gene repression by targeting and promoting the proteasomal degradation of BCL11B [63]. HIV-1 Tat abundance is affected by selective autophagy (discussed below) [64,65], and by proteasomal degradation. While lysine 48-polyubiquitination (K48Ub) of HIV-1 Tat stimulates transactivation [66,67], the K48Ub mark targets proteins for proteolytic degradation through the proteasome [68]. Similarly, the long non-coding RNA NRON also targets HIV-1 Tat to the ubiquitin/proteasome system for degradation [69].

In addition to nucleosome positioning by the BAF complex and decreased availability of HIV-1 Tat and P-TEFb, multiple epigenetic modulators involved in the post-translational acetylation, methylation, phosphorylation, ubiquitination, and crotonylation of histones, and the methylation of DNA work to silence the HIV-1 promoter [70,71]. The most studied of these are methylation and acetylation of lysine residues of histone tails that affect nucleosome stability. For instance, negatively acting NF-κB NFKB1 p50 homodimer, BCL11B, and YY1/LSF recruit HDAC1 to the 5′ LTR resulting in decreased nuc-1 acetylation, thereby increasing nucleosome stability [72,73,74]. BCL11B also recruits the histone methyltransferase SUV39H1, which catalyses the K9 trimethylation of H3 (H3K9me3) [74]. Nuc-1 is further K9 trimethylated by EHMT1, EHMT2, and LSD1 [75,76], which recruits the chromatin reader heterochromatin protein 1 (HP1) isoforms CBX1, CBX3, and CBX5 resulting in chromatin compaction. CBX5 further recruits SUV39H1 propagating the H3K9me3 mark along the HIV-1 promoter to further the heterochromatin environment, resulting in the repression of HIV-1 transcription [74].

Despite the epigenetic transcriptional elongation block, cell-associated abortive TAR element transcripts are detectable even in the absence of replication competent virus in persons receiving fully suppressive ART [9,10,77], suggesting that although viral transcription occurs during latency, downstream mechanisms that block viral RNA elongation, splicing, polyadenylation, and the nuclear export of these viral RNAs are involved in repressing HIV-1 expression [77,78]. Indeed, there is a greater block to HIV-1 transcriptional elongation, completion, and splicing than to transcriptional initiation [77,79]. Therefore, the mechanisms that control the translation, modification, and abundance of proteins involved in these steps are important regulators of HIV-1 expression. In support of this hypothesis, latent HIV-1-infected CD4^+^ T cells, the major reservoir of latent HIV-1, poorly express MATR3, an essential cofactor of HIV-1 Rev-mediated RNA export, thus decreasing translation [80], and express cellular microRNAs (miR-28, miR-125b, miR-150, miR-223, and miR-382) that potently inhibit HIV-1 production by binding to the 3′ untranslated region of mRNAs, including those of HIV-1 Tat, HIV-1 Rev, CCNT1, and components of the PBAF complex, inhibiting their translation [81,82,83,84].

## 3. Autophagy-Mediated Restriction of HIV-1

**Figure 1:**
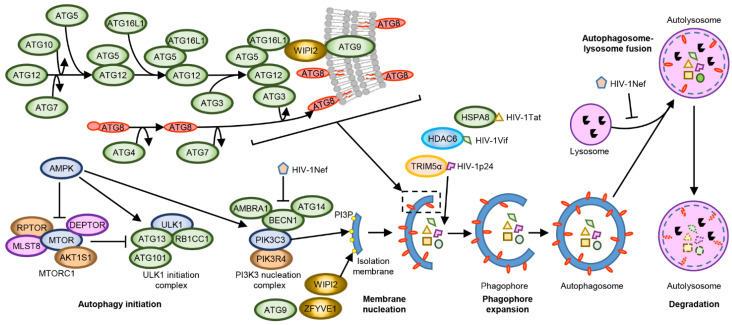
The initiation of autophagy is a multistep process. The main regulators of autophagy are MTOR, an inhibitor, and AMP-activated kinase (AMPK) an activator. MTORC1 inhibition induces autophagy through the formation of the ULK1 complex. This complex initiates the formation of the phagophore by directly activating the phosphatidylinositol 3-kinase catalytic subunit type 3 complex I (PIK3C3 complex). The PIK3C3 complex translocates to endoplasmic reticulum sites and produces phosphatidylinositol 3-phosphate (PI3P) which then recruits PI3P-binding proteins such as WIPI2 and ZFYVE1. HIV-1 Nef can inhibit this process by enhancing the association between BECN1 and its inhibitor BCL2. In the ATG12 conjugation system, ATG12 forms a covalent bond with ATG5, which then binds ATG16L1, followed by dimerization (not shown) and interaction with the PI3P-binding complex. The WIPI2b isoform acts immediately upstream of ATG16L1 and recruits ATG12–ATG5-ATG16L1 to PI3P-containing phagophores. The ATG12–ATG5-ATG16L1 complex then promotes conjugation of ATG8 family proteins with phosphatidylethanolamine (PE), which are then incorporated into phagophore membranes, where they interact with cargo receptors harbouring LC3-interacting motifs. ATG16L1, as well as the polyubiquitin-binding autophagy receptors including SQSTM1, OPTN, NBR1, and NDP52 also recruit and incorporate ubiquitin-decorated cargos into autophagosomes including HIV-1 Tat, HIV-1 Vif, and HIV-1 p24. After detachment of ATG factors, the isolation membrane closes through membrane scission, forming the autophagosome. These, then, fuse with lysosomes resulting in the degradation of the engulfed components as well as the ATG8-PE and SQSTM1 associated with the inner membrane. HIV-1 Nef affects autophagosome maturation by preventing the fusion between autophagosomes and lysosomes.

MTORC1 inhibition induces the formation of the ULK1 complex, which drives the formation of the initial autophagosomal precursor membrane structure (the phagophore) by directly activating the PIK3C3 (phosphatidylinositol 3-kinase catalytic subunit type 3) complex I (Figure 1). The PIK3C3 complex I is composed of PIK3C3, phosphoinositide-3-kinase regulatory subunit 4 (PIK3R4), Beclin 1 (BECN1), and ATG (autophagy-related protein) 14. This complex translocates to ER sites and produces phosphatidylinositol 3-phosphate (PI3P) which subsequently recruits PI3P-binding proteins, such as WIPI2 and ZFYVE1 [85]. The recruitment of these proteins results in the formation of phagophores, which expand and close through membrane scission forming autophagosomes [86]. The precise mechanisms(s) by which this occurs are not completely known. However, current research suggests that it involves two ubiquitin-like conjugation systems. The first entails the E1-like enzyme ATG7 and the E2-like enzyme ATG3 conjugating the ubiquitin-like ATG8 family proteins (microtubule-associated protein 1 light chain 3 [MAP1LC3 or more commonly LC3] A, LC3B, LC3B2, LC3C, GABARAP [GABA type A receptor associated protein], GABARAP-like [GABARAPL] 1 and GABARAPL2) to the headgroups of membrane lipid phosphatidylethanolamine (PE). The second involves the recruitment of the E3-like ATG12–ATG5-ATG16L1 complex to PI3P-containing phagophores by the WIPI2b isoform. The ATG12–ATG5-ATG16L1 complex then ligates ATG8-PE to nascent autophagosome membranes where they can interact with cargo receptors harbouring LC3-interacting regions (LIRs; a conserved sequence W/F/YxxL/I/V) [85]. ATG16L1, as well as the polyubiquitin-binding autophagy receptors including sequestosome 1 (SQSTM1, p62), optineurin (OPTN), NBR1, and NDP52, which harbour both Ub-binding domains and LIRs also recruit and incorporate ubiquitin-decorated cargos into autophagosomes [87]. After detachment of ATG factors, the isolation membrane closes and syntaxin 17 is recruited to the autophagosomes. These then fuse with lysosomes resulting in the degradation of the engulfed components as well as the ATG8-PE and SQSTM1 associated with the inner membrane [88]. As autophagy is a coordinated pathway of autophagosome formation, sequestration of autophagic cargo, autophagosome-lysosome fusion, followed by the proteolytic degradation of the sequestered cargo, dysregulation or inhibition of any step could lead to the failure of the autophagy cycle that could lead to a disturbance of cellular homeostasis leading to cell death. As an example, inhibition of autophagy by blocking lysosomal degradation can lead to the accumulation of autophagosomes, thus depleting membrane sources.

Host cells can recognise microorganism-specific pathogen-associated molecular pattern (PAMP) molecules through pattern recognition receptors (PRR). These PRR include toll-like receptors (TLR), RIG-I-like receptors (RLR), nucleotide oligomerization domain (NOD)-like receptors (NLR), and C-type lectin receptors (CLR). The most studied PRR in relation to autophagy are the TLR. Humans have 10 TLRs, each recognizing a different PAMP, and are present in many different cell types including CD4^+^ T cells and macrophages. TLRs 1, 2, 4, 5, 6, and 10 are located at the plasma membrane and recognise bacterial membrane components, whereas TLRs 3, 7, 8, and 9 are predominantly located within endosomes. The phylogenetically and structurally related TLR7/8 recognise GU-rich short single-stranded microbial RNA, such as that found in the U5 region of the HIV-1 genome [89,90]. HIV-1-Env-CD4 interactions mediates the endocytosis of HIV-1 that delivers viral genomic RNA to the endosomal TLR7/8 [91,92]. In addition to mediating the endocytosis of HIV-1, in dendritic cells, HIV-1 Env also activates and exhausts autophagy leading to a rapid shutdown of both autophagy and immunoamphisome production. This results in an increase in both cell-associated HIV-1 and transfer of HIV-1 to CD4^+^ T cells while also impairing both the innate and adaptive immune responses as autophagy interacts with the MHC-II loading machinery [93,94]. Upon ligand activation, TLR8 associates and interacts with the adapter protein MYD88 that recruits IL-1 receptor-associated kinases, leading to the NF-κB-dependent transcription of numerous pro-inflammatory mediators including IL-6, IL-12, IL-27, TNF, interferon (IFN) γ, and IL-1β in the absence of pyroptosis [95,96,97,98]. Crucially, the NLRP3-inflammasome induced secretion of IL-1β is dependent upon intact autophagy [98,99]. Similarly, exposure of plasmacytoid dendritic cells (pDCs) to infectious or non-infectious HIV-1 or to HIV-1 derived GU-rich HIV-1 RNA sequences induces IFNα production through a TLR7- and autophagy-dependent mechanism [91,100]. In addition to stimulating the release of pro-inflammatory cytokines, TLR7/8 ligation also suppresses HIV-1 replication in acute ex vivo human lymphoid tissue of tonsillar origin and renders peripheral blood mononuclear cells (PBMC) barely permissive to HIV-1 infection [101]. Further studies demonstrated that binding of TLR8 by GU-rich HIV-1 RNA sequences induces autophagy through a cathelicidin antimicrobial peptide and vitamin D-dependent mechanism [92,102], and decreases HIV-1 p24 release from HIV-1 infected macrophages through an autophagy and lysosome dependent mechanism [92,102]. Interestingly, HIV-1 downregulates IRAK4, which is essential for virtually all TLR signalling [103]. People living with HIV-1 (PLWH) have lower levels of vitamin D3 than uninfected individuals [104,105,106,107,108,109,110,111]. Additionally, the concentrations of vitamin D3 decrease during HIV-1 disease progression and correlate with increased markers of inflammation and decreased autophagy and survival rates [110,112,113]. Vitamin D3 is a known autophagy inducer, and studies show that vitamin D3 decreases HIV-1 p24 release from HIV-1 infected macrophages through an autophagy-dependent mechanism [114]. 

One of the first HIV-1 restriction factors identified was the PRR TRIM5α, which interacts with retroviral capsid proteins in the context of an intact core structure. Although TRIM5α was originally suggested to be a species-specific HIV-1 restriction factor, with rhesus TRIM5α but not human TRIM5α efficiently restricting HIV-1 infection by accelerating premature uncoating of the virus thereby inhibiting nuclear translocation and subsequent integration [115,116,117], recent literature indicates that human TRIM5α also acts as an HIV-1 restriction factor through a different mechanism. While HIV-1 binding to DC-SIGN (CD209) positive dendritic cells leads to disassociation of TRIM5α from CD209, in Langerhans cells, TRIM5α mediates the assembly of an autophagy-activating scaffold to the CLR langerin (CD207), and acts as a receptor for selective autophagic degradation of HIV-1 proteins [118,119,120,121]. Moreover, the TRIM5α-HIV-1 p24 binding promotes the synthesis of unattached K63-linked ubiquitin chains that promotes the recruitment and activation of the MAP3K7-TAB complex in an autophagy-dependent mechanism, leading to the transcription and secretion of cytokines including interferon α/β and IL-6 [118,122,123]. Interestingly, TRIM5α expression is increased in PBMC from long-term non-progressors, and this increase correlates with an increase in HIV-1 gp120 and TRIM5α co-localization in autophagic vacuoles compared with PBMC from typical progressors [124].

Although Sagnier et al. [64] suggested that autophagy selectively degrades HIV-1 Tat through a ubiquitin-independent interaction with SQSTM1, Xu et al. demonstrated that HIV-1 Tat undergoes K63-polyubiquitination, a mark recognised by SQSTM1 and the NBR1 autophagy cargo receptor [125,126,127], complexes with the CMA-specific chaperone HSPA8 and is subjected to K63Ub-selective autophagy mediated by serine hydroxymethyltransferase (SHMT) 1, SHMT2 and the BRCC36/BRISC deubiquitase complex [65].

During HIV-1 infection, the cellular restriction factor APOBEC3G restricts HIV-1 replication through the specific deamination of dCs in newly synthesised (−)-strand viral DNA, resulting in massive G-to-A invalidating hypermutations in the nascent (+)-strand viral DNA genome during reverse transcription. In addition, APOBEC3G also directly blocks reverse transcriptase elongation in a deaminase-independent manner and interferes with the integration of proviral DNA. HIV-1 Vif antagonises the APOBEC3G-mediated host defence system by recruiting APOBEC3G into the E3 ubiquitin ligase complex, thus promoting APOBEC3G degradation through the ubiquitin-proteasome pathway. However, through its C-terminal ubiquitin-binding BUZ (binder of ubiquitin zinc finger) domain, the host factor HDAC6 binds HIV-1 Vif and targets it for autophagic degradation [128]. HDAC6 is a central component of basal autophagy that promotes cortactin-dependent, actin-remodelling machinery to stimulate autophagosome-lysosome fusion and substrate degradation [129], while also facilitating the transport of misfolded proteins to the aggresome [130].

HIV-1 overcomes these innate host autophagic defences through HIV-1 Nef, which blocks autophagy initiation by enhancing the association between BECN1 and its inhibitor BCL2 in a PRKN-dependent mechanism. This blocking is done by inducing the cytoplasmic sequestration of TFEB, an upstream regulator of MTOR, and by affecting autophagosome maturation by preventing the fusion between autophagosomes and lysosomes (Figure 1) [92,131,132].

## 4. HIV-1 Cure Strategies

Although post-transcriptional regulation of HIV-1 latency is an important multifactorial process, current efforts to eradicate the latent HIV-1 reservoir pool focus on reactivating the transcription of latent HIV-1 proviruses. This approach uses latency-reversing agents (LRAs) followed by a combination of ART, with the expectation that targeted immunotherapy, host immune clearance, and HIV-1-cytolysis will be sufficient to kill latently infected cells expressing viral proteins—the “kick and kill” approach. The LRAs under investigation are various and include histone post-translational modification modulators, NF-κB stimulators, non-histone chromatin modulators, TLR agonists, and MTOR activators [133]. However, with over 160 LRA tested to date, none have shown great promise [133]. The most studied class of LRA are the histone post-translational modification modulators that include the histone deacetylase inhibitors (HDACi) givinostat, panobinostat, romidepsin, and vorinostat. Although these induce HIV-1 expression through the reversal of epigenetic silencing, they do not induce the expression of MATR3 [80], resulting in insufficiently low levels of induced viral expression to cause viral cytopathicity, or their elimination by immune effectors such as cytotoxic T cells. Unfortunately, HDACi impair cytotoxic T cell and natural killer (NK) cell functions [134,135], while also promoting the infection of uninfected CD4^+^ T cells [136].

The inhibitor of apoptosis protein (IAP) family members BIRC2 and BIRC3, are upregulated in latent HIV-1 infected cells [137,138,139,140] and are important negative regulators of non-canonical NF-κB signalling, and thus 5′ LTR-dependent HIV-1 transcription [141,142]. DIABLO/SMAC mimetics mimic the BIR-binding N-terminal tetrapeptide sequence of DIABLO, a pro-apoptogenic mitochondrial protein released into the cytoplasm in response to apoptotic stimuli to antagonise IAPs. The interaction of DIABLO/SMAC mimetics with BIRC2 and BIRC3 activates their E3 ubiquitin ligase activity promoting their autoubiquitination and proteasomal degradation [143,144,145]. Depletion of BIRC2 using DIABLO/SMAC mimetics can activate non-canonical NF-κB signalling, and can act as a LRA in latent HIV-1 cell line models [140,142,146,147,148,149,150,151]. However, only AZD5582, xevinapant, and ciapavir have shown this ability in primary cells, albeit with mixed efficacy, and none have been tested in PLWH.

Agonists against the endosomal TLR3, -7, and -9 have gone through clinical trials for LRA with mixed results [152]. In addition to this potential function, TLR agonists also enhance the generation of HIV-1-specific CD8^+^ T cells [153], while activating proinflammatory and antimicrobial responses, including the NF-κB-dependent transcription of numerous proinflammatory mediators [154] and the induction of autophagy [155]. As the hyper-activation of MTORC1 by HIV-1 Tat is necessary for HIV-1 production [25], the activation of MTOR by tideglusib, a glycogen synthase kinase 3 inhibitor, was investigated. However, although tideglusib reactivated latent HIV-1 from ex vivo primary CD4^+^ T cells derived from PLWH on suppressive ART, tideglusib, like other LRAs, failed to reverse latency in vivo [156].

**Figure 2:**
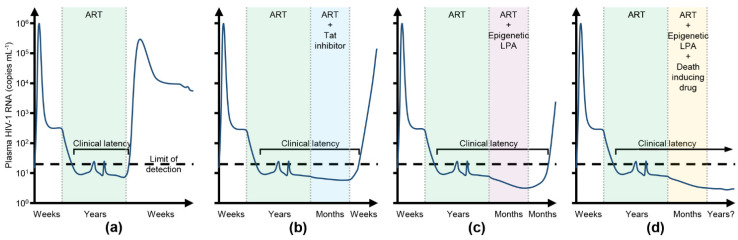
Potential strategy to achieve an HIV-1 cure. (**a**) Antiretroviral therapy (ART) suppresses HIV-1 plasma viremia, but upon interruption of ART, it quickly rebounds; (**b**) conventional HIV-1 Tat/P-TEFb inhibitors fail to silence epigenetically the HIV-1 promoter, thus when they are removed, transcription is eventually restored and HIV-1 viremia rebounds; (**c**) supplementation of ART with a latency-promoting epigenetic silencing drug (LPA) could promote a state of deep latency, but as they do not reduce the viral reservoir, rebound could occur; (**d**) supplementation of ART with both an LPA and a drug that selectively kills HIV-1 infected cells could decrease the size of the viral reservoir, significantly prolonging the time to viral rebound after ART interruption. Optimization of such a strategy has the potential to completely eliminate the viral reservoirs.

These examples highlight the overall disconnect between the promising in vitro and ex vivo results of LRAs and the poor in vivo findings. Therefore, there is an urgent need for a new approach if a functional HIV-1 cure is to be achieved. In contrast to the “kick and kill” approach, the latency promoting “block and lock” approach is designed to repress HIV-1 expression and release it to levels that can be controlled and cleared by the immune system in the absence of ART—a state of deep latency [157,158]. Deep latency is occasionally observed in some PLWH who are able to control viremia for several years after ART interruption despite possessing weak HIV-1-specific immune responses and low levels of pro-inflammatory markers [16,17,159,160]. Although these post-treatment controllers have relatively small HIV-1 reservoirs, it is theorised that a “block and lock” approach could achieve a similar transcriptionally repressive profile in people initially starting with much larger HIV-1 reservoirs, which result in normal CD4^+^ T cell counts, the absence of disease progression, and no release of replication competent virus after ART interruption (Figure 2). The “block and lock” approach has a number of advantages over the “kick and kill” approach. As the “block and lock” approach is designed to induce a state of deep latency, the safety concerns related to viral reactivation, drug resistance, and transmission associated with the “kick and kill” approach should be mitigated. Additionally, this approach could be affordable, permanent, and easily implemented in resource-limited settings. Moreover, even if the latency-promoting agents (LPA) in development fail to achieve a long-term functional cure, they could still be of clinical value as a potential new class of ART, as well as being used to uncouple HIV-1 transcription from immune system activation, enhancing clearance by the immune system.

As the control of HIV-1 Tat and P-TEFb translation, modification, turnover, and abundance are important regulators of both HIV-1 replication and latency, HIV-1 Tat, TAR RNA, and P-TEFb are all important targets for LPA development [161]. Unfortunately, the lack of specificity and/or poor pharmacokinetic properties hindered the early development of compounds targeting these proteins [162,163,164,165]. Although these early compounds decreased gross HIV-1 transcription, they had no effect on the integrated pro-virus such that when inhibition was relieved, HIV-1 viremia rapidly rebounded [166,167]. In contrast to these early attempts, an equipotent analogue of the steroidal alkaloid cortistatin A, didehydro-cortistatin A (dCA), has shown promise. dCA binds to the TAR-binding domain of HIV-1 Tat and blocks transcriptional elongation of the HIV-1 promoter [168,169], disrupting the HIV-1 transactivation positive feedback loop, while also instigating the progressive accrual of epigenetic modifications along the HIV-1 5′ LTR. This results in a repressive heterochromatin environment that epigenetically silences the HIV-1 promoter [59,170,171,172,173], even in the presence of an LRA [171]. Other recent promising LPA targeting the HIV-1 Tat/P-TEFb nexus include spironolactone [174], HEXIM1-Tat peptide [175], the BRD4 inhibitor ZL0580 [176,177], and Nullbasic, a 101 amino acid trans-dominant negative HIV-1_BH10_ Tat in which G/A residues replace the entire basic domain [178,179,180,181].

Although LPA could function to a point where HIV-1 reactivation from latency is unlikely, these drugs do not induce the destruction of the latent reservoir. Unfortunately, the larger the expressed HIV-1 reservoir, the shorter the time to viral rebound after ART interruption [182]. Therefore, combining LPA with drugs that can specifically kill latent HIV-1 infected cells could be advantageous—a “kill, block and lock” (Figure 2d). Although a number of pro-apoptotic compounds, including BCL2 antagonists, DDX3 inhibitors, PLK1 inhibitors, and RLR agonists have been assessed for their potential to specifically induce cell death of HIV-1-infected cells [183,184,185,186,187], they all either require the addition of an LRA to specifically kill HIV-1 infected cells, or induce viral transcription themselves. Thus, they are incompatible with a “block and lock” approach. As the hyper-activation of MTORC1 by HIV-1 Tat is necessary for HIV-1 production [25] and the integrity and function of both MTORC1 and MTORC2 are essential in re-activating HIV-1 from latency [26,27], the inhibition of MTOR is an attractive target for LPA development. The potent and selective ATP-competitive second generation MTOR inhibitors torkinib, torin1, and sapanisertib all repress induced basal (HIV-1 Tat-independent) and (HIV-1 Tat-dependent) HIV-1 gene expression and the reactivation of HIV-1 from in vitro and in vivo models of HIV-1 latency, as well as ex vivo primary CD4+ T cells derived from PLWH on long-term suppressive ART [25,26,188]. Further supporting the use of MTOR inhibitors, the allosteric MTORC1 inhibitors, sirolimus and everolimus inhibit the production of fully infectious HIV-1 particles from a number of different cell types while also limiting intestinal HIV-1 transmission [114,189,190,191]. Additionally, HIV-1-infected adult solid organ transplant recipients receiving these drugs better control HIV-1 replication versus patients receiving alternative immunosuppressants [192,193]. Moreover, sirolimus synergistically enhances the activity of entry inhibitors, such as vicriviroc, aplaviroc and enfuvirtide [194], and inhibits HIV-1 entry through the downregulation of CCR5 [194,195]. Although these MTOR inhibition studies did not assess the role of autophagy, the inhibition of MTOR induces autophagy. Despite being a predominantly cell survival mechanism, under certain conditions autophagy, and the proteins associated with the autophagic process, can promote cell death [196,197]. For instance, in combination with ART and ionizing radiation, sirolimus selectively kills HIV-1-infected myeloid and CD4+ T-cell reservoirs via the inhibition of viral transcription/translation and the induction of autophagy with no deleterious effect on uninfected bystander cells [198].

## 5. Autophagy as Part of a HIV-1 Cure Approach

Although HIV-1 Nef blocks autophagy initiation and autophagosome maturation, pharmacological inducers of autophagy such as sirolimus can overcome this block leading to the autophagic-degradation of HIV-1 capsid proteins and a decrease in released HIV-1 virions through an ATG5- and autophagy-dependent mechanism [114,199]. Notably, in HIV-1-infected macrophages, the LRA HDACi vorinostat, panobinostat, givinostat and romidepsin and the non-histone chromatin modulating BET inhibitor JQ1 all inhibit MTOR through a yet unidentified mechanism, leading to the dose-dependent autophagic degradation of HIV-1 capsid proteins and a subsequent decrease in HIV-1 release in the absence of cell death [200,201]. Similarly, the dual PI3K/MTOR inhibitor dactolisib (NVP-BEZ235), and the PI3K/MTOR/BRD4 inhibitor SF2523 all inhibit MTOR with similar autophagy-dependent decreases in both intracellular and extracellular HIV-1 p24 abundance in the absence of cell death [201].

Further studies demonstrated that trehalose, a naturally occurring sugar, induces MTOR-dependent and MTOR-independent autophagy in HIV-1 infected macrophages and CD4^+^ T cells, leading to the degradation of intracellular HIV-1 capsid proteins and an autophagy-dependent reduction in HIV-1 release [202,203]. Trehalose can also decrease HIV-1 entry through the downregulation of CD4^+^ expression on CD4^+^ T cells and macrophages and CCR5 on CD4^+^ T cells, thus, reducing transmission [202]. Interestingly, nelfinavir also inhibits MTOR through the upregulation of the MTOR inhibitor sestrin-2 (SESN2) resulting in autophagy induction [204]. Its role in HIV-1 restriction is yet to be pursued. Finally, the recent discovery of miRAB40, an autophagy-inducing, HIV-1-inhibiting microRNA, may also provide insights into the microRNA-mediated regulation of autophagy-mediated HIV-1 inhibition [205].

Although the autophagic degradation of HIV-1 does not have an effect on the transcriptionally latent HIV-1 provirus, autophagy can play a role in controlling latent HIV-1 infection by reducing the reservoir size through the susceptibility of latent HIV-1 infected cells to drug-induced autophagy-dependent cell death. As an example, the autophagy-inducing peptide, Tat–beclin 1, composed of the amino acids 267-284 of BECN1 attached via a diglycine linker to the protein transduction domain of HIV-1 Tat, inhibits HIV-1 replication in primary human macrophages in an autophagy-dependent mechanism [199]. This peptide also restricted in vitro replication of Sindbis virus, chikungunya virus and West Nile Virus, and reduced the mortality of mice infected with West Nile Virus and chikungunya virus through autophagy [199]. Further studies demonstrated that when encapsulated within a lipid-coated hybrid poly(lactic-*co*-glycolic acid) (PLGA) nanoparticle, the Tat–beclin 1 peptide selectively kills latent HIV-1 infected CD4^+^ T cells through Na^+^/K^+^-ATPase-mediated autophagy-dependent autosis in the absence of increased viral replication [206]. Autosis is an autophagy-dependent, non-necrotic, non-apoptotic cell death characterised by unique biochemical and morphological features. In the first phase (1a), the endoplasmic reticulum becomes dilated and fragmented, and autophagosome, autolysosome, and empty vacuole numbers increase. In the second phase (1b), the perinuclear space (PNS) swells at discrete regions surrounding the inner nuclear membrane and contains cytoplasmic materials. In the last phase (2), the morphology appears necrotic with ER, autophagosome, autolysosome numbers crashing while mitochondria and other cytoplasmic organelles swell. Also observed are a concavity of the nuclear surface and focal ballooning of the PNS, while the plasma membrane becomes porous [207]. Similar to Tat–beclin 1 nanoparticles, a lipid-coated hybrid PLGA nanoparticle encapsulated v-FLIP-α2 peptide (a ten amino acid α2-helix peptide derived from the death effector domain 1 of the K13 protein of human gammaherpesvirus 8 [208]) also induces autosis of both HIV-1-infected resting memory CD4^+^ T cells and HIV-1-infected macrophages with no deleterious effects on uninfected bystander cells, and in the absence of increased viral replication [206,209]. Nanoparticles are currently being evaluated to deliver small interfering RNAs (siRNAs) to silence gene expression in infected cells, as well as HIV-1 itself [210], and to deliver cargo(s) that directly interfere with and inhibit viral entry [211,212], or to selectively kill HIV-1-infected cells [206]. Interestingly, a biomimetic nanoparticle wrapped in a CD4^+^ T cell membrane was shown to not only neutralise cell-free HIV-1, but also reduced cell-associated HIV-1 p24 through an autophagy-dependent mechanism [213,214]. Importantly, under certain conditions, nanoparticles can be targeted to occult HIV-1 reservoir sites such as lymphatic tissue or the central nervous system (CNS) through the conjugation of cell-specific ligands to the nanoparticle surface, or with a biomimetic membrane [215,216]. Although there is currently no delivery mechanism approved to deliver RNA or peptides to occult HIV-1 reservoirs in vivo, the regulatory approval of patisiran [217], and the development and emergency use authorization of the COVID-19 mRNA vaccines BNT162b2 and mRNA-1273 [218,219] paves the way for future lipid-based nanoparticle drug research to reach sanctuary sites, including the CNS. In other studies, autophagy inducing PI3K/AKT1 inhibitors, including the clinically available miltefosine, selectively kill HIV-1 infected macrophages [220,221]. Therefore, the selective killing of HIV-1 infected cells, comprising those actively replicating virus and those within the latent reservoirs, has the potential to achieve the complete eradication of HIV-1 infection.

**Figure 3:**
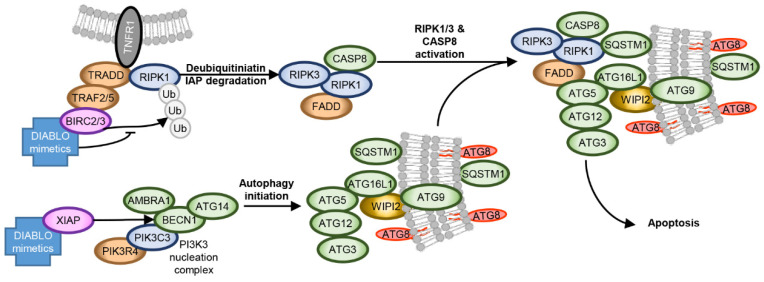
DIABO/SMAC mimetic mediated apoptosis. Treatment of latent HIV-infected cells with DIABLO/SMAC mimetics induces the degradation of XIAP and BIRC2/3, inducing autophagy, the deubiquitination of RIPK1, and the disinhibition of caspases. This leads to the formation of a death inducing signalling complex comprising of FADD, RIPK1, RIPK3 and caspase-8 on autophagosomal membranes, resulting in the cleavage and activation of caspase-8, thus enabling activation of the effector caspases (caspase-3 and caspase-7) and the execution of apoptosis.

During infection, HIV-1 increases the expression of BIRC2/3/5, XIAP, as well as TREM1 and BCL2 family members, while decreasing the expression of pro-apoptotic proteins [137,138,139,140,147,222,223,224]. In addition to inhibiting 5′ LTR-dependent HIV-1 transcription [141,142], the overexpression of IAPs plays a further important role in the establishment and maintenance of HIV-1 latency by inhibiting HIV-1-mediated cytopathogenesis. They do this through direct antagonism, ubiquitination, and neddylation of apoptosis caspases [225,226], and ubiquitination of BECN1 (reducing autophagy) and RIPK1 [227], suppressing its aberrant activation. RIPK1 is a death domain containing protein that controls differentiation and inflammation transcriptional responses [228]. The scaffolding function of RIPK1 is essential to promote pro-survival NF-κB signalling through the assembly of complex I, while activation of RIPK1 leads to either apoptosis or necroptosis [229]. RIPK1 also plays an important role in the cellular response to low energy levels and mediates AMPK-MTORC1 signalling [230]. Therefore, in addition to promoting the proteasomal degradation of BIRC2/3 and XIAP, the binding of DIABLO/SMAC mimetics to IAP also disinhibits apoptosis caspases and activates RIPK1 (Figure 3) [231]. In HIV-1-infected macrophages and latent HIV-1-infected resting memory CD4^+^ T cells, DIABLO/SMAC mimetics trigger the proteasomal degradation of IAPs that correlates with the induction of autophagy and cell death [140,147,232]. Importantly, although IAPs similarly induce the degradation of IAPs in uninfected cells, they do not undergo cell death [140,147]. The DIABLO/SMAC mimetic-mediated degradation of IAPs leads to the deubiquitination and activation of RIPK1, which in HIV-1-infected cells leads to the formation of a death-inducing signalling complex (DISC) consisting of pro-apoptotic (FADD, RIPK1, RIPK3, CASP8) and autophagy (ATG5, ATG7, SQSTM1) proteins [140,147]. Notably, inhibition of autophagy initiation, membrane nucleation, phagophore formation and expansion ablated DIABLO/SMAC mimetic-mediated apoptosis, while inhibition of autophagosome closure through membrane scission, autophago-lysosome fusion and degradation had no protective effect. Moreover, the DIABLO/SMAC mimetic-mediated DISC formation and localization to autophagosomes and resultant apoptosis required SQSTM1-mediated RIPK1 tethering to the phagophore membrane. Therefore, autophagosome membranes mediate DIABLO/SMAC mimetic-induced cell death by serving as a scaffold for efficient DISC formation rather than by autophagy degrading intracellular components. Importantly, although the DIABLO/SMAC mimetics AZD5582, xevinapant, and ciapavir are useful as LRA [142,146,148,149,150,151], treatment with birinapant, GDC-0152, and LCL-161 did not increase HIV-1 p24 antigen release indicating that the DIABLO/SMAC mimetics were killing HIV-1-infected cells in the absence of latency reactivation [140,147,232]. Notably, birinapant is also able to induce selective cell death in hepatitis B virus expressing hepatocytes [233,234,235]. Consequently, IAPs are excellent targets for therapeutic exploitation in an HIV-1 “kill, block and lock” cure strategy.

## 6. Concluding Remarks

The combination of LPA with host-directed or direct-acting autophagy and autosis inducing drugs to eliminate selectively HIV-1 latently infected cells to reduce the viral reservoir can enhance the efficacy of the “block and lock” approach, and when combined with other strategies, could potentially achieve viral eradication. However, reaching isolated body compartments, including lymph nodes and the CNS remains a major challenge to achieving the full potential of these strategies. Currently, numerous drugs have shown considerable potential in vitro and in animal models that could contribute to an HIV-1 cure. Well-designed clinical trials will be required to assess the ability of any of these approaches to penetrate and act throughout the body. As these cure strategies are evaluated, it is likely that the modulation of autophagy will be required to achieve an HIV-1 functional or complete virologic cure.
